# Brazilian women in Bioinformatics: Challenges and opportunities

**DOI:** 10.1590/1678-4685-GMB-2023-0134

**Published:** 2024-01-22

**Authors:** Thayne Woycinck Kowalski, Giovanna Câmara Giudicelli, Maria Clara de Freitas Pinho, Marília Körbes Rockenbach, Miriãn Ferrão Maciel-Fiuza, Mariana Recamonde-Mendoza, Fernanda Sales Luiz Vianna

**Affiliations:** 1 Universidade Federal do Rio Grande do Sul, Departamento de Genética, Programa de Pós-Graduação em Genética e Biologia Molecular, Porto Alegre, RS, Brazil.; 2 Hospital de Clínicas de Porto Alegre, Serviço de Genética Médica, Unidade de Genética Laboratorial, Porto Alegre, RS, Brazil.; 3 Centro Universitário CESUCA, Cachoeirinha, RS, Brazil.; 4 Hospital de Clínicas de Porto Alegre, Núcleo de Bioinformática, Porto Alegre, RS, Brazil.; 5 Hospital de Clínicas de Porto Alegre, Laboratório de Medicina Genômica, Porto Alegre, RS, Brazil.; 6 Universidade Federal do Rio Grande do Sul, Laboratório de Imunogenética, Porto Alegre, RS, Brazil.; 7 Universidade Federal do Rio Grande do Sul, Instituto de Informática, Porto Alegre, RS, Brazil.

**Keywords:** STEM, women in science, computational sciences, gender disparity, impostor phenomenon

## Abstract

Bioinformatics is a growing research field that received great notoriety in the years of the COVID-19 pandemic. It is a very integrative area, comprising professionals from science, technology, engineering, and mathematics (STEM). In agreement with the other STEM areas, several women have greatly contributed to bioinformatics ascension; however, they had to surpass prejudice and stereotypes to achieve recognition and leadership positions, a path that studies have demonstrated to be more comfortable to their male colleagues. In this review, we discuss the several difficulties that women in STEM, including bioinformatics, surpass during their careers. First, we present a historical context on bioinformatics and the main applications for this area. Then, we discuss gender disparity in STEM and present the challenges that still contribute to women’s inequality in STEM compared to their male colleagues. We also present the opportunities and the transformation that we can start, acting in academia, inside the family and school environments, and as a society, hence contributing to gender equality in STEM. Finally, we discuss specific challenges in the bioinformatics field and how we can act to overcome them, especially in low and middle-income countries, such as Brazil.

## Introduction

Over the past 25 years, advances in genome sequencing technologies associated with their decreased costs paved the way for the exponential growth of sequenced genomes (Lappalainen et al., 2019). Two decades after the completion of the Human Genome Project, genome sequencing at an individual level is becoming routine, and substantial high-throughput technologies are available and have been applied in a large variety of organisms and cell types (Chen and Coppola, 2018). With the huge amounts of data generated by these next-generation sequencing (NGS) technologies, bioinformatics has become more needed in genetics research and diagnosis laboratories (Lappalainen *et al*., 2019). Recently, bioinformatics has made great contributions during the Covid-19 pandemic in predicting the structure, origin, and evolution of SARS-CoV-2, contributing to understanding the pathogenesis of the virus and screening drugs and vaccines in record time for public health decision-making (First cases of coronavirus disease (COVID-19) in Brazil, South America (2 genomes, 3rd March 2020). The combination of bioinformatics analysis with NGS is instrumental in interpreting biological data (Chen and Coppola, 2018), such as data from genomes, transcriptomes, proteomes, epigenomes, and metabolomes.

The prodigious scale of these omics data requires storage in specialized databases (Lappalainen et al., 2019), such as the publicly available ones, which revolutionized the way of doing science. Beyond facilitating data sharing, secondary analysis of publicly available data through bioinformatic approaches is a powerful strategy to shed light on new hypotheses (Chen and Coppola, 2018), which can be later confirmed in experimental assays. With the aid of these databases, bioinformatics approaches provide a non-expensive way for identifying specific and biologically relevant targets, contributing to saving time and effort, as well as for better-allocating resources and avoiding the misuse of animal models. In low- and middle-income countries such as Brazil, alternatives for prioritizing financial investment are of great interest (Rocha et al., 2022).

Bioinformatics have been applied to several health and other science fields. However, it is a consensus that bioinformatics is largely applied in genetics and genomics. In this review for the special edition of the 60th anniversary of the Post-Graduation Program in Genetics and Molecular Biology (PPGBM), we will focus on the bioinformatics applications in genetics and we will discuss the challenges women encounter by entering this area, as well as strategies we can all commit to increase gender equality in the field. 

## Women in Bioinformatics: The past and the present

The creation of computational technologies in several areas of knowledge has led to profound changes in society at the individual and collective levels. In this context, computers and their specialized tools have become fundamental in the working toolkit of professionals and researchers in the biological and biomedical sciences. Today, almost all research projects related to those areas require bioinformatics tools, especially since the establishment of methodologies that generate a vast amount of data, such as massive sequencing technologies. Those next-generation sequencing (NGS) methods are related to a substantial change in investigations of population genetics, microbial ecology, molecular systematics, and several other areas of research (Gauthier et al., 2019).

There are controversies between the definitions of bioinformatics and computational biology, which sometimes appear to be synonymous due to the inappropriate use of the terms. For that reason, in 2000 the Biomedical Information Science and Technology Initiative Consortium (BISTIC) Definition Committee, part of the National Institutes of Health (NIH), defined bioinformatics as the *“research, development, or application of computational tools and approaches for expanding the use of biological, medical, behavioral or health data, including those to acquire, store, organize, archive, analyze, or visualize such data”* (Huerta et al., 2000). Computational biology was defined by the same committee as *“the development and application of data-analytical and theoretical methods, mathematical modeling and computational simulation techniques to the study of biological, behavioral, and social systems”*. Since the terms are very similar and sometimes incorrectly applied, many researchers not entirely familiarized with bioinformatics often believe that it is a recent area, created to meet the demand for analysis of data generated by the NGS. However, it is important to highlight that computational strategies have been applied to solve biological problems even before the Internet appears. Therefore, the history of bioinformatics started many years ago, and distinct women researchers have been important to consolidate this field.

Women pioneered the development of computer science and bioinformatics. In 1843, English mathematician and writer Ada Lovelace developed programs on Charles Babbage’s mechanical computer, contributing to the world’s first computer algorithm. As early as 1946, six mathematicians, Fran Bilas, Betty Jennings, Ruth Lichterman, Kay McNulty, Betty Snyder, and Marlyn Wescoff, developed programs for the first electronic computer in history (ENIAC) during World War II. In 1965 occurred the first attempt to systematize the knowledge of proteins, with the Atlas of Sequence and Structure of Proteins, considered the first database of biological sequences and organized by several authors. Margaret Dayhoff stands out among authors for her important role in forming bioinformatics as we know it today, both in terms of sequences and structures. She was an American physical chemist who used computational methods for her doctoral thesis in electrochemistry and realized the potential and applicability of these methods in the biological and biomedical areas. Dayhoff is considered a pioneer in the application of computation in the field of biochemistry, and she was called *‘the mother and father of bioinformatics’* by David J. Lipman, former director of National Center for Biotechnology Information (NCBI). Also in the 1960s, together with Robert S. Ledley, a physicist who used computational resources in biomedical problems, Dayhoff developed Comprotein, a computer program to determine the primary structure of proteins. Dayhoff also developed the one-letter amino acid code to simplify the manipulation of protein sequence data that was stored on punched cards. Furthermore, she developed one of the first substitution matrices and contributed significantly to the development of phylogenetic studies. In the following decades, 1970s and 1980s, advances allowed the analysis of complete genomes. While in the 1990s-2000s, the use of the internet associated with next-generation sequencing led to the generation of a large volume of data and the proliferation of bioinformatics tools (Gauthier et al., 2019).

In the current scenario, women continue to contribute significantly to bioinformatics. For example, Emmanuelle Charpentier and Jennifer A. Doudna jointly won the 2020 Nobel Prize in Chemistry. They received the award for their research into the development of Clustered Regularly Interspaced Short Palindromic Repeats (CRISPR), a genome-editing method that has revolutionized the biological and biomedical sciences for its potential therapeutic use in genetic diseases. Although the CRISPR itself is commonly related to the wet lab, this technique could never be done without the help of bioinformatics tools that previously help the scientist to choose the sequences that should be removed from the genetic material (Alkhnbashi et al., 2010). This was the first time that the Nobel Prize was given to two women together. Also in 2020, the Brazilian researchers Jaqueline Goes de Jesus and Ester Cerdeira Sabino joined the team responsible for mapping the genome of the coronavirus in Brazil (de Jesus *et al*., 2020). Furthermore, the biologist Ana Tereza Ribeiro de Vasconcelos and her team identified a new variant of the SARS-CoV-2 in Rio de Janeiro. She is responsible for the bioinformatics laboratory at the Laboratório Nacional de Computação Científica (LNCC), where she has worked since 1984. Vasconcellos is considered one of the pioneers in bioinformatics in the country. In 1998, she organized the formation of a “national genome network”, with the training of 25 laboratories in 15 Brazilian states. Among other contributions, she was also one of the creators and first president of the Brazilian Association of Bioinformatics and Computational Biology (AB3C). 

The Brazilian mathematician Maria Emília Machado Telles Walter was also a pioneer in bioinformatics. She is a professor at the Department of Computational Science at the Universidade de Brasília (UnB), working mainly with genome sequencing and rearrangement, and comparative genomics. Professor Glória Regina Franco is also an important Brazilian bioinformatician. She is a professor at the Department of Biochemistry and Immunology at the Universidade Federal de Minas Gerais (UFMG), and her studies are mainly dedicated to genomics, transcriptomics, and proteomics of the neglected disease schistosomiasis, caused by *Schistosoma mansoni*, although she also works with other organisms. More recently, the bioinformatician and computer scientist Raquel Cardoso de Melo Minardi has played a prominent role in the dissemination of bioinformatics and training human resources in this area. She is a professor at the Department of Computer Science at the Universidade Federal de Minas Gerais (UFMG), working on the development of algorithms and computational tools for biological and health issues. Since 2018, Minardi has maintained the project “OnlineBioinfo Bioinformática”, a YouTube channel that aims to produce content for teaching and scientific dissemination on bioinformatics, in addition to training students capable of acting in scientific dissemination on the subject. Minardi works so that women know about and have access to science and technology courses so that they can pursue a career in bioinformatics, which is very important to disclose to women the different possibilities of working in bioinformatics applications.

## Gender disparity in STEM

Considering the great applications of bioinformatics and that advances in high-throughput technologies will continue to rise concomitantly with a large amount of genome-sequenced information, the support of powerful bioinformatics tools and qualified professionals will become more and more necessary to face it (Counsell, 2003). The areas encompassed by bioinformatics, science, technology, engineering, and mathematics (STEM) have been widely dominated by male researchers. Being bioinformatics relatively new, the following sections will address the gender disparity issue in the STEM field as a whole. Nevertheless, all the challenges and perspectives addressed here are present in the daily routine of a female bioinformatician.

Female representation in the STEM areas is frequently affected by gender stereotypes and gender bias (Roper, 2019). Even though in biological sciences women received around 60% of bachelor degrees, in math and physics, men represent 60%, while in engineering and computer science, the difference is even bigger, with only 20% of female representation (Bowman et al., 2022). Not only in universities the numbers are uneven, but also in the professional career, in which women receive fewer credits and stimulation to develop, the numbers are more disparate (The Lancet Digital Health, 2020). Even though the number of women choosing to graduate in STEM has been increasing over time, when it comes to employed females in these areas, numbers are still uneven. For instance, half of science and engineering graduates are women in the United States (US), but less than 30% of them work in the area; in the United Kingdom, women are less than 25% of science employees (Roper, 2019). One reason for these unbalanced numbers is the stereotype that girls and women are less interested or skilled in STEM, mostly engineering or computer science, which is disseminated early and impacts the number of young girls pursuing a degree in this area (Master et al., 2021). In addition to gender stereotypes, sexual harassment and the remuneration gap between men and women are also related to the reasons women decide not to continue to work and research in the STEM areas (The Lancet Digital Health, 2020). Moreover, women who continue their careers in this area receive fewer opportunities in leadership positions, such as in academic informatics programs. For example, between 2017 and 2019, men represented the majority of leadership in the US (3/4) (Griffin et al., 2021). Furthermore, authorship in the research environment is also affected by the disparities. According to the Gendermetrics platform (In Life Science, Multidisciplinary, Earth & Environmental and Chemistry field), around 1/3 of the first authorship and co-authorship are represented by women, and less than 20% of the last authors were women (Bendels et al., 2018). The Covid-19 pandemic also highlighted gender inequities in the STEM and medicine (STEMM) environment, such as women with children reporting a decreased number of hours dedicated to work (Krukowski et al., 2022). For instance, a study in the US showed that in a STEMM faculty during the pandemic, women’s productions as first authors or coauthors decreased significantly, but men remained the same (Krukowski *et al*., 2021).

In Brazil, women tend to seek education more than men, but they have more difficulties finding a job (Organisation for Economic Cooperation and Development (OECD), 2019). Furthermore, in the research area, even though the number of women’s publications is increasing in the past years, they receive lower-level funding from the governmental foment agency Brazilian National Council for Scientific and Technological Development (Conselho Nacional de Desenvolvimento Científico e Tecnológico, CNPq), while men receive higher amounts (Valentova et al., 2017). Leadership position disparities are also present, as an example, in the Brazilian Society of Neuroscience and Behavior (SBNeC), from 1978 to 2020, only two women were presidents, and the majority of board posts were occupied by men (Erthal et al., 2021). In authorships of scientific publications, the inequality remains present. For instance, in a Brazilian Surgical Journal, 72.2% of the authors were men and according to a study, in two cardiology journals, around 70% of the last authors were male (Mesquita et al., 2022).

The gender gap is slowly decreasing over the years, but women still are a minority in the STEM fields (Valentova et al., 2017). Some approaches like a support for female scientists throughout motherhood, specific funding and hiring not only of women but also other minorities, focusing on diversity, as well as stimulating the interest of young girls in pursuing a career in STEM might be helpful to change that scenario and continuingly diminish the inequality (The Lancet Digital Health, 2020; Krukowski et al., 2022).

## Challenges and opportunities

Gender inequality in STEM cannot be explained by only looking at the academic environment. Several cultural aspects, stereotypes, and economic issues are involved in explaining women being underrepresented in the STEM areas. Here, we highlight and discuss the main challenges to overcome gender inequality focused on the academic area, and a solution for each matter is presented in Table 1.

**Table 1 -:** Challenges and solutions regarding gender equality in STEM.

Challenge	How to solve it?
The stereotype threat	Do not replicate the STEM male stereotype. When in contact with a child, remember that boys and girls might be equally interested in a math problem, a scientific experiment, or a computer’s architecture.
The gender-biased education and work-family conflict	Fight against stereotypes from early childhood to empower young girls. Stimulate gender-sensitive education and gender diversity, promote flexible work arrangements and equal pay for women and men.
The impostor phenomenon	To encourage diversity in STEM by hiring females and minorities, asserting their job is important and relevant as male’s.
The cultural paradigm	Organizations and educational institutions can encourage girls and women through mentorship programs, creating a supportive and inclusive environment.
The holes in the leaky pipeline	Recruitment and retention will only work by obliterating any type of discrimination and creating a more inclusive environment in the STEM field. Any form of sexual harassment or gender/racial discrimination must have a zero tolerance institutional policy.
The lack of the female role model	Investment on professionalizing and leadership programs specific for women, also involving other minorities (i.e., racial, LGBTQIAP+) to increase relatability with the new students.

### The stereotype threat

STEM stereotypes are present in several niches in society, not only in academic environments. From offensive jokes to controversial studies, women are raised to believe women lack STEM ability when compared to men because of biological differences (i.e., brain anatomy, hormones), but none of them was proven (Girelli, 2023). This existing stereotyped STEM male model is replicated through generations of parents and/or educators (Tomasetto et al., 2011). Throughout women’s lives, beliefs that STEM fields are male territory are more intricate than we acknowledge. The math male stereotype exemplifies this issue. Mathematics is represented in social media as a cold, pervasive, more masculine area (Girelli, 2023), whilst other careers, such as pedagogy and language arts, are presented as more feminine (Chaffee and Plante, 2022). As reviewed by Girelli (2023), the math male stereotype might negatively impact the performance of both genders because it influences the expectation and self-concepts of each individual ability (Girelli, 2023). Boys might choose a STEM career because they are grown up believing language arts are not proper for men (Chaffee and Plante, 2022). Women who chose mathematics might underperform in tests because of the fear they might endorse the male math stereotype if they do not reach high scores on those tests (Girelli, 2023). The stereotype threat is a challenge that can only be solved by addressing gender inequity (Table 1). Gender inequity is considered the root for several issues regarding women’s mental health in academia, including the impostor phenomenon.

### The gender-biased education and the work-family conflict

Gender bias can be defined as a favoritism to one gender over another, related to proper roles, behaviors, and expectations for men and women. It is commonly associated with a negative connotation for women, especially because of our patriarchal society concepts (Alves et al., 2020). From birth, children are confronted with gender stereotypes, such as the room decoration and colors of their clothes (blue for boys and pink for girls), as well as toys associated with boys and girls. Children are exposed to gender bias mainly by their parents/educators, who were also previously shaped and influenced by conducts imposed by society according to their gender (Alves *et al*., 2020). Endendijk et al. (2013) conducted a study with 355 three-years-old children and their families as part of the longitudinal study *Boys will be Boys?*, aiming to evaluate how their gender stereotypes influence the socialization and development of children in the first four years of life. The authors found that mothers and fathers differ in parental attitudes related to gender stereotypes, with mothers acting more implicitly and fathers more explicitly. These differences could be related to intentional or unintentional attitudes that influence how children should act according to their gender. 

An important consequence of gender-biased education is the work-family conflict. Greenhaus and Beutell (1985) evaluated sources of conflict between work and family roles and suggested that the major subjects that contribute to work-family conflict are time-based, strain-based, and behavior-based. The authors, however, did not mention how this differently impacts men and women. They mentioned that women in professional positions probably work enough hours to negatively impact her husband’s work responsibilities, once he will have to participate in more family activities. However, it’s important to recognize that married employee couples are actually part of two distinct careers. Also, young girls are commonly raised to motherhood and homework, which contributes to excluding women from knowledge-power spaces and affects their mental health (Narvaz et al., 2013). Other subjects to the work conflict for women are related to time, such as extensive and extra working hours, and raising children (Vilela and Lourenço, 2018). The balance between women’s professional occupation and their family roles are directly related to gender-biased education. It is also related to the fact that despite the increasing number of women working in traditional male-dominated professions, gender segregation persists within the workplaces (Lee et al., 2013), which could discourage women from working on STEM careers.

In this context, how parents/educators act in front of gender bias is particularly important to shape the identity of young children (Finco, 2013). For example, the socialization processes between parents and children related to the exposure to gender-typical different toys are important to assure the optimal development and character formation of the children (Boe and Woods, 2018). In this sense, the impact of fairy tales stories should also be debated. In general, those stories presented women with a submissed attitude, mainly restricted to homemaking activities, while men are considered strong and superior, and are usually involved in adventures (Alves et al., 2020). Also, parents usually believe that science careers are more interesting for boys than girls (Tenenbaum and Leaper, 2003). With that, young girls are teached to act with passive behaviors and are not stimulated to be scientists, and this explains, at least in part, why we have more men in STEM careers (Diekman et al., 2015). These examples highlight the importance of fighting against stereotypes from early childhood, so children could grow up understanding the importance of gender equality (Table 1). This is especially important to empower young girls and encourage them to enter and stay in STEM areas (Botton and Strey, 2018).

### The impostor phenomenon

In 1978, two women, Clance & Imes, addressed the subject of female professionals who achieved impressive academic positions and, even then, believed they did not deserve such accomplishment (Clance and Imes, 1978). This self-doubt belief was named the “impostor phenomenon” (IP) by the authors, and has since then, been popularly addressed as the “impostor syndrome”. The authors evaluated white women, observing behaviors such as diligence and hard work, described as common in IP (Clance and Imes, 1978). IP is not infrequently combined with burnout because, for those who are experiencing IP, working hard avoids being discovered as a fraud (Mullangi and Jagsi, 2019). Added to that, perfectionism, self-efficacy, and anxiety have also been associated with IP (Chakraverty, 2022). 

More recently, studies have focused on understanding the environmental contribution to IP development. The current scenario includes workplace harassment, racial discrimination, a hypercompetitive environment, and the underrepresentation of women and racial minorities in high academic positions (Chakraverty, 2022). To diminish IP, profound cultural changes and nourishment of women’s careers are needed (Table 1). However, Mullangi and Jagsi (2019) point out that IP is only a symptom (consequence) of a disease (gender inequity), being gender inequity the real challenge to be solved. And for that, there is an urgent need to address the cultural beliefs, discussing educational gender disparity in different environments. Despite efforts in academia, gender-biased education might start in school and at home.

### The cultural paradigm

We previously described how the impostor phenomenon, stereotype threat, gender-biased education, and work-family conflict are challenges for women. However, it is important to highlight that racial and cultural inequities make these factors even more difficult for trans women and non-white women. We need to recognize that, as suggested by the Cell Editorial Team, *science has a racism problem* - and we need to fight against this (Cell Editorial Team, 2020). Through literature, we can observe implicit biases in judgment related to the individual’s skin color and ethnicity (Calaza et al., 2021). Artes (2018) described that access to graduation and postgraduation in Brazil is differentiated by gender and race, and the worst indicators are related to the black population. Also, it is important to maintain a conscious connection with scientific perspectives that are non-hegemonic and non-eurocentric to encourage dialogue between differences and question discourses that reinforce discrimination and stereotypes (Benite et al., 2018). In STEM careers, black women work harder to be seen as competent when compared to white women, and they have to face more complex stereotypes and stigmas (Pietri et al., 2018). Nguyen et al. (2021) showed in their study the importance of empowering black girls and women by illustrating moments of inspiration to overcome obstacles and promote their capacity. 

STEM areas are also harder for trans women. The challenges are mostly related to discrimination, lack of representation, and access to resources, leading to an underrepresentation. Supporting and promoting the visibility of trans women in STEM is important to create a more equitable scientific community (Restar and Operario, 2019) (Table 1). Scientists with different backgrounds could be interested in different research questions, therefore embracing diversity is related to the innovation caused by their distinct perspectives. Hofstra et al. (2020) showed that underrepresented groups produce higher rates of scientific novelty. Also, this study highlights that inequality and injustice is perpetuated in science, and therefore the scientific community needs to create and embrace efficient policies that promote gender and racial/ethnicity equity to contribute to a more equal academic research environment and avoid problems such as the leaky pipeline.

### The holes in the leaky pipeline

The “leaky pipeline” is a metaphor that tries to explain the gender disparities in STEM, pointing out a flaw in the recruitment and retention of women in academia. Female representation in STEM is not only reduced relative to male, but it is also more unlikely and slower for women to achieve a leadership position (Diekman et al., 2015). Many aspects regarding the social issue of gender inequality have already been discussed here. This section will focus on the academia work environment and the reasons behind the leaky pipeline. The academic environment is described as chilly, unwelcoming, and threatening, a perception that is more prominent to women than men, because the hostile and uncomfortable environment is driven towards women (Casad et al., 2021). Although legal protections have helped to address hideous behaviors and attitudes, it is undeniable women and other minorities face subtle discriminations in their workplaces, leading to greater stress and diminished performance (O’Brien et al., 2016). Exclusion, incivility, sexual harassment, and discrimination are some situations perceived more often by female STEM members (Casad *et al*., 2021). A study performed in the University of Michigan with STEM professionals and students demonstrated that 30% of the women interviewed reported sexual harassment, whilst only 4% of the men had suffered from it (Jagsi et al., 2016). Sexual harassment and gender discrimination have a negative impact on a scientist productivity, whilst a nonsexist work environment has been positively associated to increased job satisfaction and academic success (Jagsi *et al*., 2016). In order to fix the leaky pipeline it is not only necessary to work on recruitment and retention. Small institutional actions can be applied to increase gender equality and diversity in STEM. From workspace decoration to informative institutional communication, the work environment in STEM can be more inclusive, favoring elements that might absorb women and other minorities (Casad *et al*., 2021) (Table 1). The best way to address sexual harassment and discrimination is by applying a zero-tolerance policy (Carr et al., 2019). In addition, mentoring and sponsorship, especially having a successful female example, can help improve women retention in the STEM field. 

### The lack of a female role model

Women in STEM have a tendency to publish less papers than men, not only due to the harassment suffered and the work-family conflict, but also because they receive less supervision than their male colleagues (Van Oosten et al., 2017). However, women engage in a range of time-consuming and crucial tasks in academia, yet these responsibilities often go unrecognized and therefore are not reflected by the commonly adopted evaluation metrics and criterias. Hence, research on STEM gender inequality has focused on the importance for girls to have a female role model, in order to persist in a STEM career (Cheryan et al., 2017). Although it is difficult to measure the impact of a role model in a student’s career, the lack of a successful female example is believed to increase gender disparity in STEM (Cheryan et al., 2017). As an example, Daldrup-Link (2017) points out the lack of objectivity in which women are criticized (many times by male colleagues) for talking too loud or being intimidating; criteria used for this misjudgement are often subjective and not based on scientific ideas or contributions of the female worker. Many young scientists who receive such treatment disengage from their purposes, leaving academia, or even developing mental issues; it is not uncommon to see these girls become bitter or resentful women, imposing a barrier for new students and reinforcing the male-dominant environment (Daldrup-Link, 2017).

Female leadership is underrepresented in the STEM areas. A study performed in the United States has demonstrated that around 50% of the STEM students are female, however most of them will never be qualified for a leadership role (Daldrup-Link, 2017). And those who reach these positions are less likely to be supported by their own institutions than their male colleagues (Van Oosten et al., 2017). This lack of mentoring results in limited collaborations and isolation from the rest of the department, which is considered an implicit bias on women career advancement (Casad et al., 2021). On this matter, Van Oosten *et al*. (2017) exemplify a leadership lab program, which must include strategies to recognize the women’s value in STEM and develop their professional skills. In addition, the authors mention the need to understand the impact of being a woman in a male-dominated field. Finally, the concept of relatability must also be reinforced when talking about a female role model. Relatability refers to models with whom the students identify themselves, feeling a deeper sense of similarity and identification (Lockwood and Kunda, 1997). Here, the impact of the female role model was brought into perspective (Table 1). However, gender is not the only source of relatability in STEM. The lack of these role models might help to explain other types of underrepresentation, such as racial minorities, with most of female scientists being white (Abraído-Lanza et al., 2022). 

## The Brazilian bioinformatic challenge

There are several specific challenges in the bioinformatics field that can be explained by the gender disparities discussed above. Although both computer and biological sciences women can migrate to bioinformatics, it is established that the majority of the professionals (male or female) in bioinformatics have a biological science background (Counsell, 2003; Rocha et al., 2022). The integration of these areas is the root of bioinformatics; it must also be present, to avoid mistaken codes or algorithms and association data without biological sense. However, in this scenario, bioinformatician women have to cross other boundaries, including the intimidating terminal full of command lines. It is not an easy task because, as it has been discussed, gender disparity is more expressive in computational sciences than even other STEM areas (Bonham and Stefan, 2017). The solution, however, is similar to the other STEM fields: gender equality must be addressed from childhood, encouraging girls to be computational scientists with the same heartiness as boys.

Counsell (2003) also discusses the difficulties to teach bioinformatics because of the students lack of previous computation basic skills. This scenario is worsened in low and middle-income countries, with less courses, workshops, and financial support than high-income countries (Moore et al., 2021). In Brazil, it is necessary to mention that digital inclusion is also unequal, even among health professionals (de Moraes et al., 2009), and computational infrastructure is not always ideal (Rocha et al., 2022). In addition, it is estimated only 5% of Brazilians are fluent in English, the language of the majority of bioinformatics tools and databases; however, as well as in any other educational scenario, gender inequality prevails, with more Brazilian men being fluent in English than women (British Council, 2014). The lack of digital inclusion and of English fluency smite Brazilians from several professions, not only women and not only in STEM. However, in spite of the lack of studies in the area, the stereotype threat, the gender-biased education, and the cultural paradigm, might affect more women than their male colleagues in regard to computational and English skills.

To better comprehend the insertion of Brazilian female scientists into the bioinformatics field, an analysis in the Bioinfo Brasil dashboard was conducted. According to the dashboard, 254 scientists are listed in Graduate Programs in Bioinformatics, comprising 150 male and 54 female scientists, an approximate 1:3 (female:male) ratio. There are several female relevant names, such as forecited Raquel Minardi; however, the latest manuscripts and bioinformatics tools developed by the Brazilian bioinformaticians are mainly published with a male scientist as first author and a male researcher as corresponding/last author (for further information, please access: https://lubianat.github.io/bioinfo_brasil/dashboard. This data demonstrates the Brazilian challenge in Bioinformatics is similar to what is encountered in other countries.

## Women in Bioinformatics: the future

To solve the gender inequality in bioinformatics, or in the STEM field as a whole, is not an easy task because it requires intervention in different environments, such as family, school, and even cultural habits (Verdugo-Castro et al., 2022). Scientific information on equal gender ability and psychological measures to address girls’ self-confidence and self-efficacy can help to diminish the myths on male STEM ability (Verdugo-Castro *et al*., 2022) and to empower women to denunciate any type of harassment or discrimination. Salary equity and institutional programs to favor mentoring and female leadership are urgently needed in order to reduce gender disparity (Abraído-Lanza et al., 2022). A breaking point for women in bioinformatics, dissimilar than the other STEM fields, is the opportunity to construct the area with more gender equality. Although there are still several social and cultural habits that are challenging to perpass, topics such as inclusion and diversity are much more debated today than in the time of the expansion of computer science or other STEM fields. There are more tutoring programs and opportunities even for girls and women that live in more vulnerable scenarios. And despite literature being scarce, it is reasonable to affirm that the future of bioinformatics is optimistic. Nevertheless, we must discuss the reality of gender inequality as a society to transform the paths of several bioinformatician women.
